# Crystal structure and thermoelectric properties of Sr–Mo substituted CaMnO_3_: a combined experimental and computational study†
†Electronic supplementary information (ESI) available. See DOI: 10.1039/c5tc02318a



**DOI:** 10.1039/c5tc02318a

**Published:** 2015-11-13

**Authors:** D. Srivastava, F. Azough, R. Freer, E. Combe, R. Funahashi, D. M. Kepaptsoglou, Q. M. Ramasse, M. Molinari, S. R. Yeandel, J. D. Baran, S. C. Parker

**Affiliations:** a School of Materials , University of Manchester , Manchester , M13 9PL , UK . Email: Robert.Freer@manchester.ac.uk; b National Institute of Advanced Industrial Science and Technology , Midorigaoka , Ikeda , Osaka 563-8577 , Japan; c SuperSTEM Laboratory , SciTech Daresbury Campus , Daresbury WA4 4AD , UK; d Department of Chemistry , University of Bath , Claverton Down , Bath BA2 7AY , UK . Email: S.C.Parker@bath.ac.uk

## Abstract

A combination of experimental and computational techniques has been employed to study doping effects in perovskite CaMnO_3_. High quality Sr–Mo co-substituted CaMnO_3_ ceramics were prepared by the conventional mixed oxide route. Crystallographic data from X-ray and electron diffraction showed an orthorhombic to tetragonal symmetry change on increasing the Sr content, suggesting that Sr widens the transition temperature in CaMnO_3_ preventing phase transformation-cracking on cooling after sintering, enabling the fabrication of high density ceramics. Atomically resolved imaging and analysis showed a random distribution of Sr in the A-site of the perovskite structure and revealed a boundary structure of 90° rotational twin boundaries across {101}_orthorhombic_; the latter are predominant phonon scattering sources to lower the thermal conductivity as suggested by molecular dynamics calculations. The effect of doping on the thermoelectric properties was evaluated. Increasing Sr substitution reduces the Seebeck coefficient but the power factor remains high due to improved densification by Sr substitution. Mo doping generates additional charge carriers due to the presence of Mn^3+^ in the Mn^4+^ matrix, reducing electrical resistivity. The major impact of Sr on thermoelectric behaviour is the reduction of the thermal conductivity as shown experimentally and by modelling. Strontium containing ceramics showed thermoelectric figure of merit (*ZT*) values higher than 0.1 at temperatures above 850 K. Ca_0.7_Sr_0.3_Mn_0.96_Mo_0.04_O_3_ ceramics exhibit enhanced properties with *S*_1000K_ = –180 μV K^–1^, *ρ*_1000K_ = 5 × 10^–5^ Ωm, *k*_1000K_ = 1.8 W m^–1^ K^–1^ and *ZT* ≈ 0.11 at 1000 K.

## Abstract 2

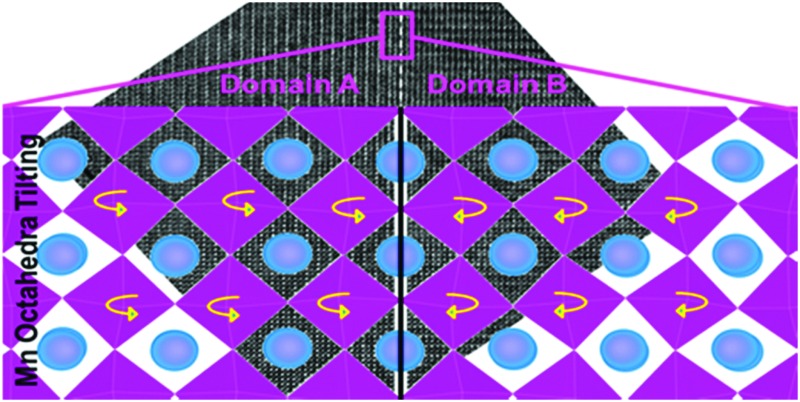
Experimental and modelling investigation of Sr/Mo (co-)doped CaMnO_3_ highlighted the role of Mn^3+^ and presence of domain boundaries in thermal transport and thermoelectric properties.

## Introduction

Thermoelectric materials are being engineered to improve the efficiency of generators that can produce electrical energy from waste heat. For over half a century, semiconductors such as Bi and Pb based tellurides have been used in niche applications.^1^ Thermoelectric oxide materials have attracted increasing attention in last two decades since the report of a high power factor in NaCo_2_O_4_.^2^ Oxides offer distinct advantages of high temperature and chemical stability which opens a wide range of new applications. The efficiency of TE materials depends on the magnitude of the dimensionless figure of merit (*ZT*); *ZT* = *S* ^2^ *σT*/*k* where *S* is the Seebeck coefficient, *σ* the electrical conductivity, *T* is the absolute temperature, and *k* the thermal conductivity. Various cobaltites, including Ca_3_Co_3_O_9_, Ca_2_Co_2_O_5_ and Bi_2_Sr_2_CoO_2_ have already been reported with *ZT* value as high as 1.2.^3–5^ The perovskites SrTiO_3_ and CaMnO_3_, and also ZnO have been identified as promising n-type thermoelectric materials.^6,7^

CaMnO_3_ is an antiferromagnetic insulator at 300 K and stabilizes in the orthorhombic *Pnma* structure with unit cell parameters ∼√2*a*_p_ ∼2*a*_p_ ∼√2*a*_p_, where *a*_p_, is unit cell length of the ideal cubic perovskite.^8,9^ Using high-temperature X-ray diffractometry and thermal analysis Taguchi *et al.* ^10^ reported the existence of two high temperature phase transitions from room temperature orthorhombic to tetragonal at 896 °C and from tetragonal to cubic symmetry at 913 °C. Using *in situ* electron diffraction, Bocher *et al.* ^11^ reported only one transition from orthorhombic to cubic on heating for CaMn_0.98_Nb_0.02_O_3_.

CaMnO_3_ possesses a relatively high Seebeck coefficient (–250 μV K^–1^), but the electrical conductivity, *σ*, is too low (10 to 100 S m^–1^ in the temperature range 300–1000 K) resulting in a low power factor (*S* ^2^ *σ*) for the un-doped material. Un-doped CaMnO_3_ also shows a relatively high thermal conductivity of 3.5–2.5 W m^–1^ K^–1^ in the temperature range 300–1000 K. The power factor of CaMnO_3_ has been enhanced by A-site substitution with different lanthanides.^12–14^ Similarly, an improved power factor has been achieved by B-site substitution with heterovalent cations including Nb, Ta, W, Ru and Mo through introducing Mn^3+^ in the matrix.^10,15–21^ In a study of the effect of Nb and Ta, Xu *et al.* ^15^ reported a lower resistivity and Seebeck coefficient for Nb doping, achieving a *ZT* of 0.08 at 1000 K for CaMn_0.96_Nb_0.04_O_3_. Later, Bocher *et al.* ^16^ investigated the thermoelectric properties of CaMn_1–*x*_Nb_*x*_O_3_ (*x* ≤ 0.08). They prepared the ceramics by both conventional mixed oxide and chemical routes; the reported TE data for the mixed oxide samples are in the same range as the data reported by Xu *et al.* ^15^ However, significantly improved TE properties were reported for the chemically prepared samples; the composition with *x* = 0.02 showed a *ZT* value of 0.2 at 900 K, increasing sharply from 0.2 to 0.3 at 1000 K. The main reason for the improved *ZT* was the remarkable reduction of thermal conductivity of the chemically prepared samples to around 0.8 W m^–1^ K^–1^ in the measured temperature range compared to thermal conductivity values of 2.8 W m^–1^ K^–1^ at 300 K to 1.5 W m^–1^ K^–1^ at 1000 K for the mixed oxide samples.

Miclau *et al.* ^17^ showed that heterovalent doping by W at the B site leads to generation of Mn^3+^ in the matrix, resulting in the reduction of the electrical resistivity and Seebeck coefficient giving an overall improvement in the power factor. The effect of W substitution on the thermal conductivity was not reported.

Recently, Thiel *et al.* ^18^ studied the system CaMn_1–*x*_W_*x*_O_3_ (*x* ≤ 0.05), employing a chemical route similar to that used by Bocher *et al.* ^16^ They found that W substitution increased the power factor with small reduction in the thermal conductivity achieving a *ZT* of 0.15 at 1000 K. The *ZT* value further increased to 0.25 at 1225 K. This increase in *ZT* from 1200 K to 1225 K was reported to be due to the loss of oxygen and creation of oxygen vacancies. A reduction in *ZT* above 1225 K was attributed to a phase transition from orthorhombic to cubic symmetry.

The effect of oxygen stoichiometry on the crystal structure has been investigated by several groups. Reller *et al.* ^19,20^ and Loshkareva *et al.* ^21^ suggested that ordered super structures are formed by the introduction of oxygen vacancies. It was later shown that the application of tensile strain lowers the formation energy of oxygen vacancies selectively, depending on the lattice site, which prompted a possible new route to engineering vacancy ordering in epitaxial thin films.^22^ Ordering of oxygen vacancies was also found to strongly affect the thermoelectric properties of CaMnO_3_. Using *ab initio* calculations, Molinari *et al.* ^23^ found that partially reduced structures exhibited more favourable Seebeck coefficients compared to the highly reduced structures, while electrical resistivity was highly dependent on the vacancy ordering. However none of the simulated oxygen deficient structures showed enhanced power factor and *ZT* values compared to stoichiometric CaMnO_3_. In parallel and independently, the computational observations of the effect of oxygen content by Molinari *et al.* ^23^ were confirmed experimentally by Schrade *et al.* ^24^ They showed that oxygen deficiency reduced the absolute value of Seebeck coefficient and reduced the electrical conductivity.

Taguchi *et al.* ^25^ showed that replacing up to 50% of the A site ions in CaMnO_3_ by Sr had a marked effect on the structure, the electrical resistivity (up to 973 K) and Seebeck coefficient. Upon increasing the strontium content the lattice parameters increase, but there is no change in the orthorhombic symmetry; the Mn–O–Mn bond distances are independent of the Sr content but Mn–O–Mn bond angles increase with the increasing Sr content. Electrical resistivity increases with the Sr content, whereas the Seebeck coefficient decreases with increasing Sr. It was suggested that the changes in the transport properties are influenced by the Mn–O–Mn bond angles.

Recently, Pi *et al.* ^26^ explored the effect of low levels of substitution of Mo for Ca on the thermoelectric properties of CaMnO_3_. They observed an increase in electrical conductivity and reduction in the Seebeck coefficient. For samples containing 4 mol% Mo, *i.e.* CaMn_0.96_Mo_0.04_O_3_, the Seebeck coefficient was –110 μV K^–1^, the thermal conductivity was 3.5 W m^–1^ K^–1^, and the *ZT* was 0.01 at room temperature. Earlier, Maignan *et al.* ^27^ studied the effect of higher levels (up to 15 mol%) of Mo substitution. They found the room temperature structure was orthorhombic up to 12 mol%, but transformed to monoclinic symmetry at higher levels of Mo. The room temperature electrical conductivity is ≈100 S m^–1^ for most compositions but the room temperature thermal conductivity is greatly influenced by Mo substitution, reducing from 2.8 W m^–1^ K^–1^ (7 mol% Mo) to 1.4 W m^–1^ K^–1^ at 15 mol% Mo.

For Sr–Mo co-doped CaMnO_3_, structural, microstructural and thermoelectric characterizations are mainly limited to low temperatures, up to 300 K. Okuda and Fujii^28^ investigated the properties of Ca_1–*x*_Sr_*x*_Mn_0.98_Mo_0.02_O_3_ (*x* = 0.00 to 0.75). They reported the structure to be orthorhombic (*pnma*) up to *x* = 0.5, which transforms to tetragonal (*I*4/*mcm*) at *x* = 0.75. In agreement with the work of Taguchi *et al.*,^25^ Okuda and Fujii^28^ found that Sr substitution enlarges the Mn–O–Mn bond angle, which facilitates electron transfer of the electrons injected by the Mo substitution. Okuda and Fujii^28^ suggested that the combined effects of one electron transfer and disorder in the A-sites (caused by Sr substitution) increases electron transport which enhances the thermoelectric figure of merit. A thermoelectric figure of merit *ZT* up to 0.03 was reported for Ca_0.75_Sr_0.25_Mn_0.98_Mo_0.02_O_3_ at room temperature.

Based on the promising low temperature thermoelectric properties of Sr–Mo co-doped CaMnO_3_, we investigated the crystal structure, microstructure and high temperature thermoelectric properties of Sr–Mo co-doped CaMnO_3_. Advanced electron microscopy in conjunction with atomistic calculations has been employed with a view to establishing correlations between structural characteristics and thermoelectric properties.

## Experimental methodology

Ceramic samples of Ca_(1–*x*)_Sr_*x*_Mn_(1–*y*)_Mo_*y*_O_3_ with *x* = 0, 0.1, 0.3, 0.6 and *y* = 0.02 and 0.04 (listed in Table 1) were prepared by the conventional mixed oxide route.

**Table 1:** Ceramic compositions

Number	Formulation	Sample code
1	CaMn_0.96_Mo_0.04_O_3_	CMM4
2	Ca_0.9_Sr_0.1_Mn_0.98_Mo_0.02_O_3_	C9S1MM2
3	Ca_0.9_Sr_0.1_Mn_0.96_Mo_0.04_O_3_	C9S1MM4
4	Ca_0.7_Sr_0.3_Mn_0.96_Mo_0.04_O_3_	C7S3MM4
5	Ca_0.4_Sr_0.6_Mn_0.96_Mo_0.04_O_3_	C4S6MM4

Starting materials were high purity powders of CaCO_3_ (Solvay, 99.5%), SrCO_3_ (Solvay, 99.5%), MnO_2_ (Sigma Aldrich, 99.9%) and MoO_3_ (Sigma Aldrich, 99.9%). MnO_2_ was wet milled for 24 h in a vibratory mill using zirconia balls and propan-2-ol in order to reduce the particle size. The powders were weighed in batches according to the required formulations and wet milled for 24 h in a vibratory mill using zirconia balls and propan-2-ol. The powders were then dried at 85 °C for 24 h and calcined twice at 1100 °C for 4 h. The calcined powders were attrition milled using zirconia balls and distilled water for 8 h and then freeze dried. The powders were uniaxially compacted into pellets of 20 mm diameter and 5 mm thickness at a pressure of 50 MPa prior to sintering at 1200–1450 °C for 4 h in air. Cooling rates of 6 °C h^–1^, 60 °C h^–1^, 180 °C h^–1^, and 480 °C h^–1^ were used.

Densification was assessed by the Archimedes method. Structural analysis was undertaken by X-ray diffraction using a Philips PW1830 system operating at 50 kV and 40 mA. The samples were first ground flat using 400 grade SiC and then scanned from 10–100° 2*θ* in steps of 0.05° with a dwell time of 20 s per step. Rietveld analysis of the data was undertaken using TOPAS 4.2.^29^

The microstructures were examined by scanning electron microscopy (Philips XL30 FEGSEM equipped with EDX capability). The samples were ground using 240, 400, 800 and 1200 grade SiC and polished using 6 μm and 1 μm diamond paste. The final polishing stage employed an oxide polishing suspension (OPS).

The samples for TEM and STEM investigation were prepared by both ion beam thinning and crushing techniques. For ion beam-thinning, the specimens were first ground on 1200 grade SiC to reduce the thickness to ∼300 μm. They were ultrasonically cut into 3 mm diameter disks (Model KT150; Kerry Ultrasonic Ltd) and then dimpled (Model D500; VCR Group, San Francisco, CA) to reduce the thickness of the center of the disk to 30 μm. Finally, the disks were ion beam thinned (using a Gatan precision ion polishing system model 691; PIPSTM) operating at 4–6 kV. For the crushing method, the sintered disks were crushed to powders using an agate mortar and pestle. Grains of individual powders were dispersed in chloroform, dropped onto a copper grid with a holy carbon film, and then dried. The structures were initially investigated using selected area electron diffraction (SAED) and high-resolution transmission electron microscopy (HRTEM) techniques using a FEI FEGTEM (Tecnai G2, Hillsboro, OR) operating at 300 kV. Subsequently, atomic-resolution structural characterization was carried out using a dedicated aberration-corrected Nion Scanning Transmission Electron Microscope (Nion UltraSTEM100, Nion Company, Kirkland, WA) located at the Daresbury SuperSTEM Laboratory in the United Kingdom.

The Seebeck coefficient and electrical conductivity were measured simultaneously using a ULVAC ZEM 3 in a helium atmosphere. Thermal conductivity was obtained by determining density (Archimedes method), thermal diffusivity (laser flash technique; facility built in house – argon atmosphere) and heat capacity (Netzsch STA 449C; nitrogen atmosphere); thermal conductivity was calculated from the product of all three parameters.

## Computational methodology

DFT and potential based techniques were applied to a range of compositions Ca_*x*_Sr_1–*x*_MnO_3_. The formulations are listed in Table 2.

**Table 2:** Formulations for *ab initio* calculations

Number	Formulations	Symbol
1	CaMnO_3_	CMO
2	Ca_0.75_Sr_0.25_MnO_3_	C7.5S2.5MO
3	Ca_0.5_Sr_0.5_MnO_3_	C5S5MO
4	Ca_0.25_Sr_0.75_MnO_3_	C2.5Sr7.5MO
5	CaMnO_2.75_	CMO2.75O
6	CaMnO_2.5_	CMO2.5O
7	Ca_0.6875_Sr_0.3125_Mn_0.9375_Mo_0.0625_O_3_	C0.7S0.3M0.9M0.1O

The DFT methodology was successfully used in our previous study on the effect of oxygen sub-stoichiometry on the thermoelectric properties of CaMnO_3–*δ*_.^23,30^ However, a brief summary is given here for completeness.

The structural models generated to determine the effect of Sr substitution on Ca, comprised four CaMnO_3_ units with G-type antiferromagnetic order. Increasingly, Ca, was substituted by aliovalent Sr to form compositions of Ca_0.25_Sr_0.75_MnO_3_, Ca_0.5_Sr_0.5_MnO_3_ and Ca_0.75_Sr_0.25_MnO_3_, to address the effect of Sr substitution on the thermoelectric properties.

The structural model generated to determine the effect of Mo substitution on Mn, comprised 16 CaMnO_3_ units with G-type antiferromagnetic order, which enabled simulation of Ca_0.6875_Sr_0.3125_Mn_0.9375_Mo_0.0625_O_3_. The optimized unit cell was a 2 × 2 × 2 expansion of the *Pnma* 5.4116 Å × 7.6339 Å × 5.3952 Å with *α* = *β* = *γ* = 90 degree cell.

A combination of planewaves and all-electron codes was used. For all models, geometry optimization was performed using spin-polarized generalized-gradient approximation (GGA) and the projector augmented wave (PAW) approach as implemented in the VASP code.^31,32^ The exchange correlation functional applied was the PBE with the inclusion of the Hubbard *U* term using the Liechtenstein approach (*U* = 5 eV and *J* = 1 eV). All calculations employed 3D boundary conditions, a cutoff energy for the planewave basis of 550 eV and convergence criteria of 0.001 eV Å^–1^ on the forces for ionic relaxation and 10^–8^ eV per atom for electronic relaxation. The Brillouin zone was sampled using 6 × 6 × 6 and 2 × 2 × 2 Monkhorst–Pack grids to ensure convergence for the 4 CaMnO_3_ and 16 CaMnO_3_ unit simulation cells, respectively. The electronic partial density of states (PDOS) for the Ca_0.6875_Sr_0.3125_Mn_0.9375_Mo_0.0625_O_3_ structure was evaluated using the GGA+U. The electronic structures for the 4 CaMnO_3_ unit simulation cells were calculated using the full-potential linearized augmented plane wave besides the local orbitals (L/APW+lo) method as implemented in the WIEN2k code.^33,34^ The muffin-tin radii were set to 2.18, 2.09, 1.84 and 1.63 Bohr for Sr, Ca, Mn and O, respectively, and the energy convergence criterion was set to 10^–5^ Ry. A mesh of 4896 *K*-points in the irreducible wedge of the Brillouin zone was chosen to generate high quality band structures. The electronic transport calculations were evaluated by solving the semi-classical Boltzmann transport equation within the constant relaxation time (*τ*) approximation as implemented in the BoltzTraP code.^35^ The choice of relaxation time was 0.5 × 10^–16^ s as chosen by Molinari *et al.*,^23^ and successfully applied to other oxide thermoelectric materials.^36,37^

Two methodologies were used to calculate the lattice component of the thermal conductivity (*k*_lattice_). In the case of DFT calculations of CaMnO_3_ systems, *k*_lattice_ was evaluated for all the compositions within the PBE+U using the Phono3py code.^38–40^

However, for the system containing the grain boundaries, due to the size of the system, potential based calculations were carried out using a potential model developed by Teter,^41^ based on partial charged rigid ions. The potential parameters employed are listed in Table 3.

**Table 3:** Teter potential parameters,^41^ *A*_ij_, *ρ*_ij_ and *C*_ij_, for the Buckingham form, *φ*_ij_ = *A*_ij_ exp(–*r*_ij_/*ρ*_ij_) – (*C*_ij_/*r*_ij_^6^) representing the interactions between ions in CaMnO_3_. Partial charges are associated with the atomic symbol

Interactions	*A*_ij_ (eV)	*ρ*_ij_ (Å)	*C*_ij_ (eV Å^6^)
Ca^1.2+^–O^1.2–^	7747.1834	0.252623	93.109
Mn^2.4+^–O^1.2–^	73547.649	0.190091	226.25
O^1.2–^–O^1.2–^	1844.7458	0.343645	192.58

Energy minimization employed the METADISE code;^42^ it shows that the potential model can reliably and accurately represent the experimental structure of CaMnO_3_ (Table 4). Generation^43,44^ of the domain boundary {101}, generally described as rotation twins across {101}_orthorhombic_, was made by rotating one CaO terminated {101} surface 180 degrees relative to a fixed surface. Energy convergence was tested and no variation was seen upon a three-fold increase of the number of species.

**Table 4:** Experimental and simulation structural parameters

	*a* (Å)	*b* (Å)	*c* (Å)	*α* = *β* = *γ* (degree)
Exp. (*Pnma*)^8^	5.2816	7.4573	5.2675	90
Calc. (*Pnma*)	5.2649	7.4589	5.2582	90

Molecular dynamics simulations were carried out using the LAMMPS code^45^ on a 10 240 CaMnO_3_ unit simulation cell (146.99 Å × 59.87 Å × 59.89 Å). Two systems were simulated; one consisting of a stoichiometric single crystal CaMnO_3_ and the other containing two domain boundaries spaced approximately 75 Å apart. The domain boundaries were perpendicular to the *x* direction and the boundary plane was in the *yz* plane. The simulation cell was relaxed in the *NPT* ensemble at 500 K until the fluctuation of the volume was minimized and the lattice vectors were set to their average values. This was followed by a 20 ns *NVT* simulation at 500 K with a timestep of 1 fs to calculate the thermal conductivity. The Green–Kubo^46,47^ method was used. The heat flux of the system was calculated at intervals of 10 timesteps over the 20 ns simulation and then correlated at each time interval and in each dimension. The correlations were integrated using the trapezoidal rule to yield thermal conductivities as a function of the integral length. A comprehensive estimation of the error is challenging^48^ and so we chose to calculate the error on the thermal conductivity from the size of the fluctuation of the integral.^49^

## Results and discussion

### Physical properties

The effect of particle size of starting powders and calcination processing was examined. It was found that by reducing the average particle size (*d*_50_) of the MnO_2_ starting powder to 1–2 μm and repeating the calcination step at 1100 °C, a single phase perovskite could be obtained. This subsequently improved homogeneity of ceramics on sintering.

All the undoped CaMnO_3_ samples contained cracks when sintered to densities above 88%. Crack-free samples with densities of at least 90% were obtained by substituting Sr for part of Ca, replacing part of Mn by Mo and using a slow cooling rate of 180 °C h^–1^ after sintering (sample densities: C9S1MM2 = 90%, C9S1MM4 = 90%, C7S3MM4 = 94%, and C4S6MM4 = 93%). Faster cooling rates than 180 °C h^–1^ led to cracking; slower cooling rates led to no significant improvement in sample performance. Thus, 180 °C h^–1^ was adopted as the standard cooling rate for all samples.

### Phase analysis

**Fig. 1:**
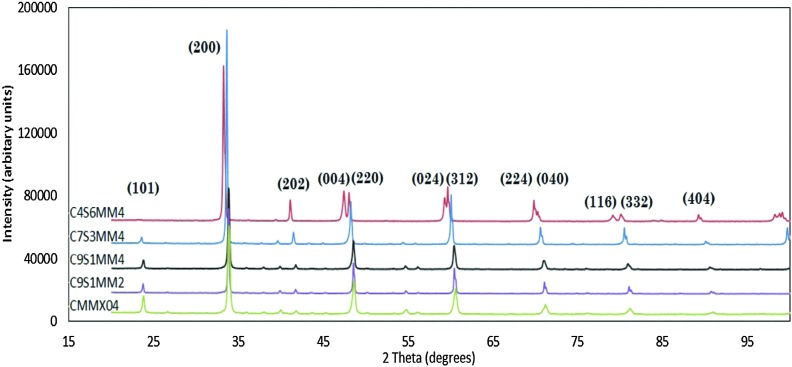
Room temperature, X-ray powder diffraction spectra of Ca_1–*x*_Sr_*x*_Mn_1–*y*_Mo_*y*_O_3_ ceramics.

Fig. 1 presents the X-ray diffraction spectra for all the formulations (Reitveld refinements of the spectra are provided in ESI†). All the spectra could be refined on the basis of a perovskite structure. With the exception of C4S6MM4, all the reflections can be indexed as orthorhombic perovskites, similar to that for un-doped CaMnO_3_^7,8^ with space group *Pnma*, where *a*_orthorhombic_ ≈ *c*_orthorhombic_ ≈ *a*_cubic_√2 and *b*_orthorhombic_ ≈ 2*a*_cubic_. The X-ray diffraction spectrum for C4S6MM4 can be refined in a tetragonal symmetry with *I*4/*mcm* space group, with *c*_tetragonal_ ≈ *a*_cubic_ and *c*_tetragonal_ ≈ *a*_cubic_. The observation of a change of symmetry upon increasing the Sr content is consistent with the findings of Okuda and Fujii.^28^ This change of room temperature symmetry from orthorhombic to tetragonal by substitution of Sr suggests that Sr widens the transition temperature window in CaMnO_3_ which is only 18 °C in the undoped end-member material.^9^ This widening of the temperature window for the symmetry change allows sufficient time (under conditions of slow cooling) for atomic rearrangement in the ceramic during cooling after sintering, thus preventing cracking. Hence, the incorporation of Sr doping effectively helps to modify the phase transformation temperature window, which with the controlled cooling rate, enables the preparation of dense, crack-free samples.

The XRD spectra show that individual peaks are displaced to lower values of 2Theta as Sr substitution is increased, reflecting the increase in lattice parameters as a result of substitution of species of higher ionic radii: Sr^2+^ (0.144 nm) for Ca^2+^ (0.134 nm) in cuboctahedral coordination and Mo^6+^ (0.059 nm) for Mn^4+^ (0.053 nm) in octahedral coordination. The experimentally-determined, refined structural parameters for all compositions are shown in Table 5; the residuals from the refinement (*R*_wp_/GOF) are sufficiently small to have confidence in the data. For comparison purposes, the calculated structural parameters for selected Ca_1–*x*_Sr_*x*_MnO_3_ compositions, evaluated using *ab initio* techniques, are shown in Table 6.

**Table 5:** Experimentally-determined, refined structural parameters for ceramics of Ca_1–*x*_Sr_*x*_Mn_1–*y*_Mo_*y*_O_3_ at room temperature

		CMM4	C9S1MM2	C9S1MM4	C7S3MM4	C4S6MM4
Space group		*Pnma*	*Pnma*	*Pnma*	*Pnma*	*I*4/*mcm*
*R*_wp_/GOF		2.54/1.88	3.40/1.91		2.99/2.12	4.16/3.12

Lattice parameters						
*a* (Å)		5.3029	5.3013	5.3074	5.3369	5.3465
*b* (Å)		7.4915	7.49505	7.5019	7.5372	—
*c* (Å)		5.2918	5.2999	5.3158	5.3351	7.6522
*V* (Å)^3^		210.2311	210.5856	211.64	214.601	218.739

Ca/Sr						
	*X*	–0.0351	–0.03250	0.0288	–0.02894	0.0000
	*Y*	0.2500	0.2500	0.25	0.2500	0.5000
	*Z*	0.0068	0.0070	–0.008	–0.00251	0.2500
	*b*_eq_	0.42	0.45/4	1.484/0.3104	0.1/0.50	0.89
	Occupancy	1	0.9/0.1	0.9/0.1	0.7/0.3	0.4/0.6

Mn/Mo						
	*X*	0.5000	0.5000	0.5	0.5000	0.0000
	*Y*	0.0000	0.0000	0.0	0.0000	0.0000
	*Z*	0.0000	0.0000	0.0	0.0000	0.0000
	*b*_eq_	1/0.42	0.5/0.05	0.4265/0.319	3/0.2	0.42/1
	Occupancy	0.96/0.04	0.98/0.02	0.96/0.04	0.96/0.04	0.96/0.04

O_ap_						
	*X*	0.5113	0.5106	0.489	0.4742	0.0000
	*Y*	0.2500	0.2500	0.25	0.2500	0.0000
	*Z*	–0.0665	–0.0661	0.067	0.0628	0.2500
	*b*_eq_	0.40	1.5	1	2.249	0.42
	Occupancy	1	1	1	1	1

O_eq_						
	*X*	0.2879	0.2877	0.285	0.2877	0.2187
	*Y*	–0.03410	–0.0330	0.033	–0.0250	0.2813
	*Z*	–0.2893	–0.2883	0.711	–0.2150	0.0000
	*b*_eq_	0.32	1.6	1	2.4	0.24
	Occupancy	1	1	1	1	1

**Table 6:** Calculated structural parameters for different Ca_1–*x*_Sr_*x*_MnO_3_ compositions evaluated using *ab initio* techniques

Phase	Symbol	*a* (Å)	*b* (Å)	*c* (Å)	*α* = *β* = *γ* (degree)
CaMnO_3_	CMO	5.366	7.534	5.311	90
Ca_0.75_Sr_0.25_MnO_3_	C7.5S2.5MO	5.378	7.589	5.361	90
Ca_0.5_Sr_0.5_MnO_3_	C5S5MO	5.394	7.639	5.401	90
Ca_0.25_Sr_0.75_MnO_3_	C2.25S7.25MO	5.417	7.665	5.440	90

**Fig. 2:**
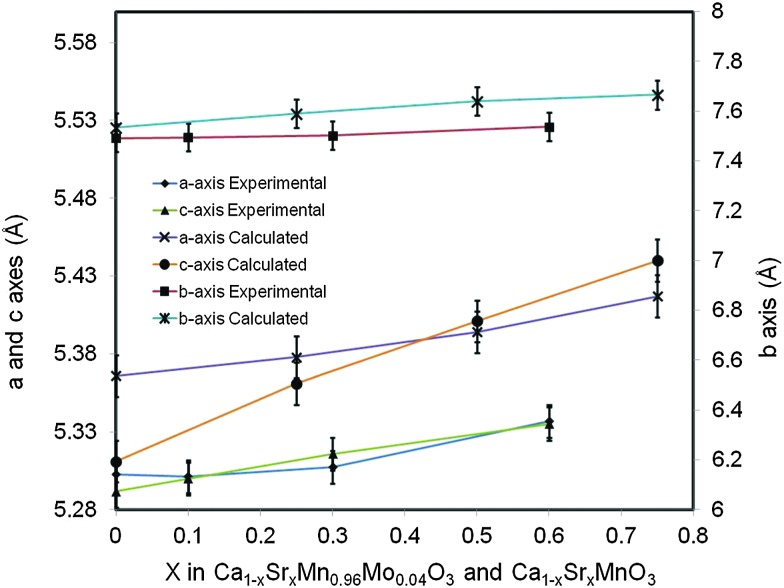
Lattice parameters for Ca_(1–*x*)_Sr_*x*_Mn_0.96_Mo_0.04_O_3_, experimental compositions and Ca_(1–*x*)_Sr_*x*_MnO_3_ computational compositions as a function of the Sr content.

The change of lattice parameters as a function of the Sr content for both experimental compositions and compositions used in *ab initio* calculations is shown in Fig. 2. In both cases the lattice parameters increase with the increasing Sr content, in very good agreement with data reported by Taguchi *et al.* ^25^ As highlighted above this is due to larger ionic radii of Sr^2+^ compared to that of Ca^2+^. The *ab initio* lattice parameters are generally overestimated compared to the experimental values; this is a well know consequence of the methodology used (GGA+U).

### Electron microscopy

**Fig. 3:**
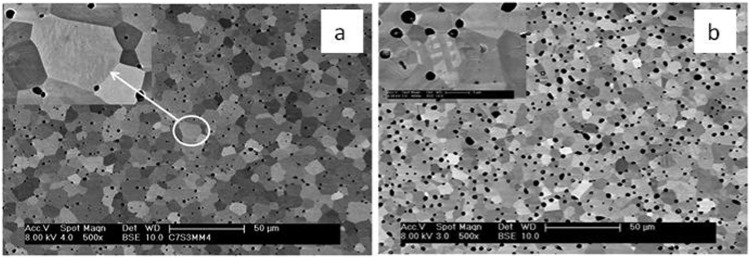
Back scattered SEM images of C7S3MM4 and C4S6MM4.

The details of the microstructures were examined by SEM. Backscattered electron SEM images of C7S3MM4 and C4S6MM4 are shown in Fig. 3. The grains are equiaxed in shape with narrow grain size distributions; most individual grains are in the 5–10 μm range. Porosity is located at the grain boundaries and also trapped within the grains. For both compositions all the grains exhibit sub-grain boundaries arising from the transition from the high temperature cubic form to the low temperature orthorhombic or tetragonal form.^9,10,28^ An enlarged image of a grain of each composition (top left insets of Fig. 3a and b) shows details of the sub-grain boundaries. The morphology of the sub-grain boundaries is very different; for C7S3MM4 the boundaries are predominantly wavy in shape, whilst for C4S6MM4 the boundaries are straight. This is due to differences in their low temperature forms of symmetry – being orthorhombic for C7S3MM4 and tetragonal for C4S6MM4 (Table 1). In view of identification of significant differences in sub-grain features by SEM, more detailed microstructural investigations were undertaken by HRTEM and STEM-based electron energy loss spectroscopy (STEM-EELS).

**Fig. 4:**
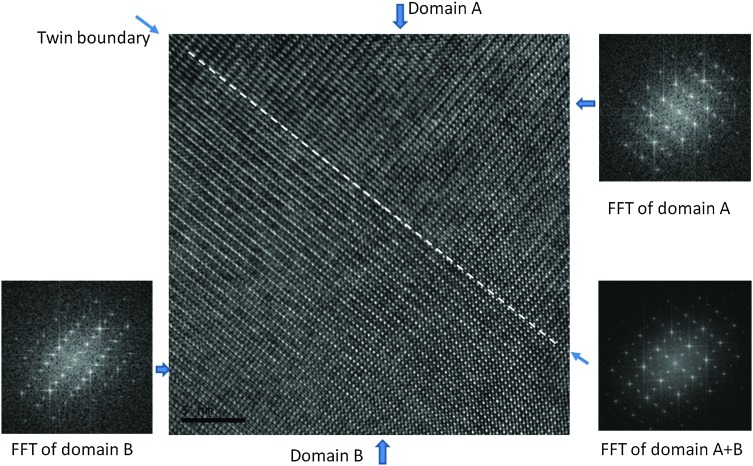
{101} HRTEM image of the twin domains A and B in C7S3MM4. On the top right and bottom left of the HRTEM image are the Fourier Transform (FFT) for domain A and domain B, respectively. The FFT from the twin boundary area is shown in the bottom right of the HRTEM image being a superposition of FFT in individual domains.

TEM studies gave a better insight into the nature of the sub-grain features observed in SEM. Fig. 4 illustrates a HRTEM image of the boundary which was frequently observed in C7S3MM4 ceramics. The Fourier Transform (FFT) for domain A and domain B and the twin boundary is shown in the figure. The FFT for both domains can be indexed as 〈101〉 orthorhombic zone axes which are rotated at 90° to each other to form the twin boundary. This type of twin boundary has been frequently observed in orthorhombic perovskites^50,51^ and is described as rotation twins across {101}_orthorhombic_. As highlighted above, the features are symmetry-breaking transition induced boundaries. These sub-grain twin boundaries may contribute, by additional phonon scattering, to the low thermal conductivity of CaMnO_3_ compared to that of other perovskites such as SrTiO_3_.^52^

To further understand the materials, a detailed study of the distribution of Sr and Ca in the A-site, the structure of the twin boundary and the valance state of Mo, the samples C7S3MM4 and C4S6MM4 were examined by aberration corrected STEM at atomic resolution.

**Fig. 5:**
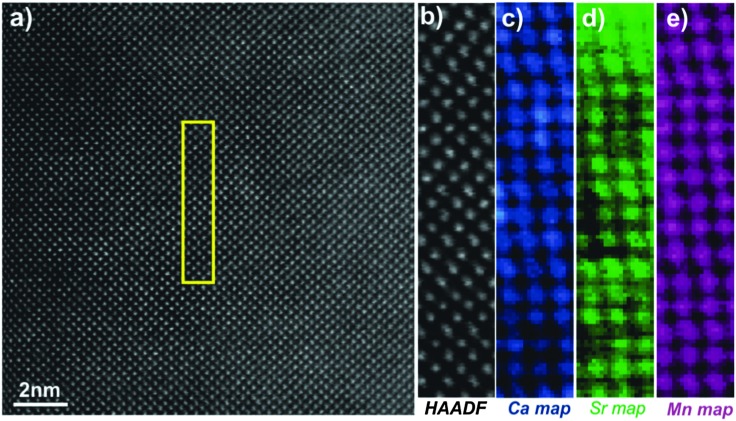
[010] zone axis HAADF-EELS data for C7S3MM4. (a) HAADF image, (b) HAADF image acquired simultaneously during EELS data acquisition, (c) Ca EELS map, (d) Sr EELS map, (e) Mn EELS map.

**Fig. 6:**
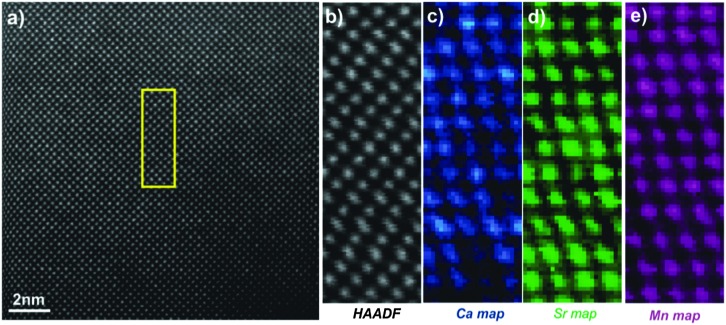
[001] zone axis STEM-EELS data for C4S6MM4: (a) HAADF image, (b) HAADF image acquired simultaneously during EELS data acquisition, (c) Ca EELS map, (d) Sr EELS map, (e) Mn EELS map.

Fig. 5 shows the [010] atomic resolution and elemental chemical maps for ceramic C7S3MM4. The area used for elemental mapping is marked with a yellow square in Fig. 5a. The HAADF signal during the EELS acquisitions and maps of Ca, Sr and Mn are presented in Fig. 5(b–e). These maps were generated by integrating the intensity of the Ca, Sr and Mn L_2,3_ edges over a 40 eV window above the respective edge onsets, after subtraction of the decaying background using a power law model. The atomically resolved maps show the random distribution and absence of any ordering of Sr and Ca in the A-site of the perovskite structure. Similar data were collected for ceramic C4S6MM4 along the [001] orientation as shown in Fig. 6. It was found that Ca/Sr distribution is also random for this formulation.

**Fig. 7:**
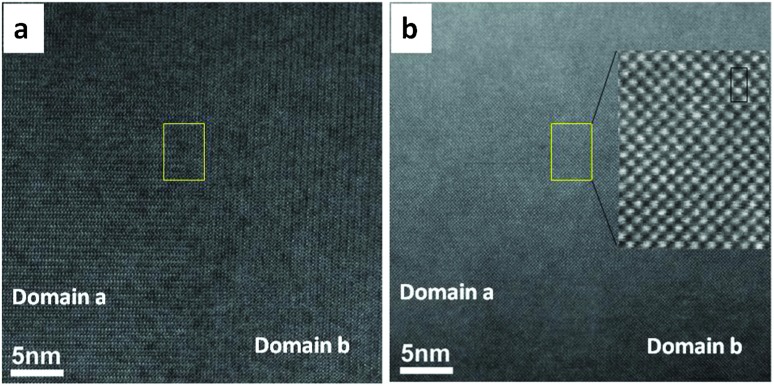
(a) Aberration corrected bright field image (BF) STEM image of the twin boundary, (b) corresponding HAADF of the twin boundary, the enlarged HAADF image of a part of twin boundary is shown in the right side of the image. The rectangle in each figure represents the unit cell.

In order to observe the structure of the twin boundary and relate it to the thermoelectric properties, aberration-corrected high-angle annular-dark-field (HAADF) and bright field (BF) images were collected as shown in Fig. 7. The absence of any displacement of A-sites (Ca and Sr) or B-sites (Mn and Mo) columns in the HAADF images as shown in the enlarged section of Fig. 7a suggests that the different BF contrast is related to the change in the oxygen positions for the two [101]_ortho_ and [–101]_ortho_ planes.

**Fig. 8:**
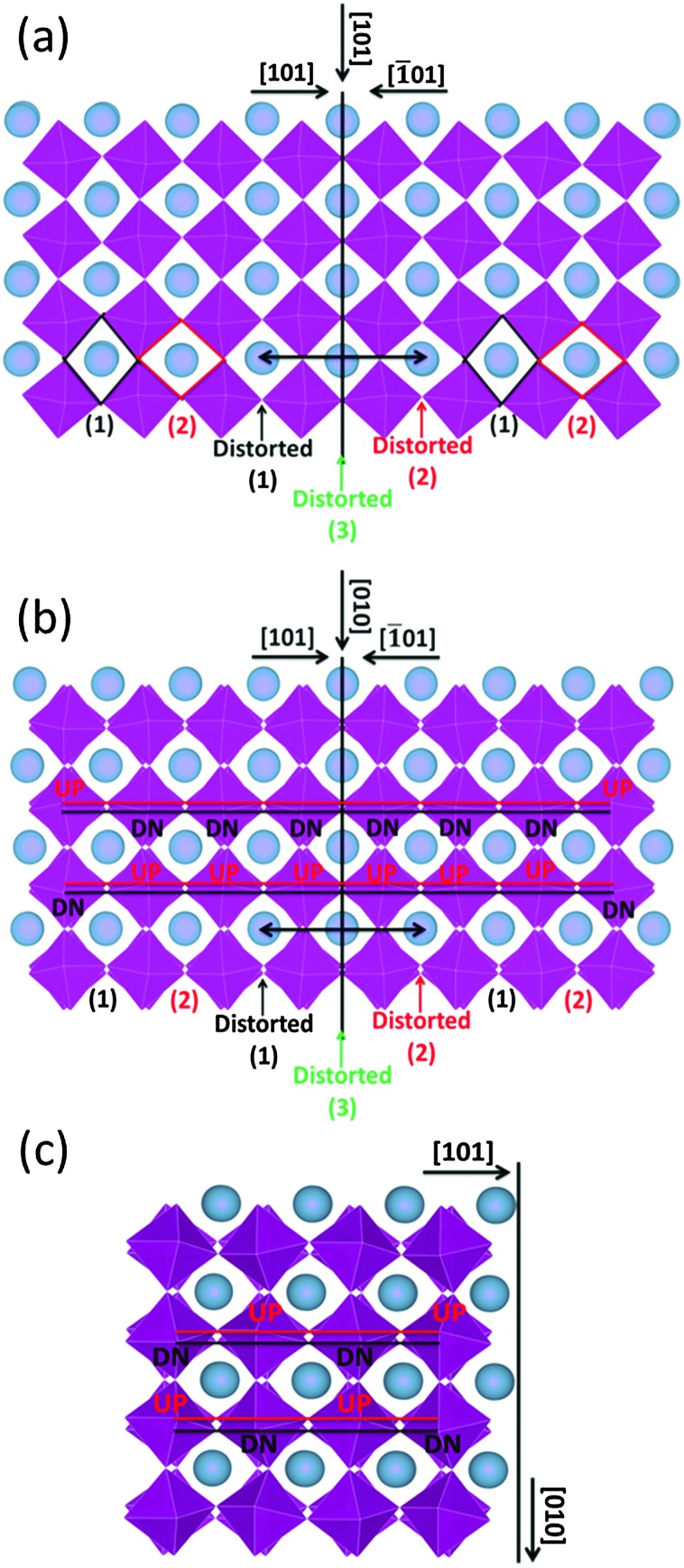
Schematic representation of the domain twin boundary (a) along the [101] and (b) [010] directions, based on energy minimization calculations. For comparison (c) depicts the {101} surface. Blue spheres are A-site cations and purple octahedra are B-site octahedra.

The formation energy of the domain twin boundary was evaluated using energy minimization simulations; a very low value of 0.10 J m^–2^ was calculated. Thus, this bulk-like boundary is thermodynamically easy to form as it is formed by joining two bulk regions with oppositely oriented Mn octahedra. Contrary to experimental approaches and images, simulation techniques can identify the position of oxygen atoms (Fig. 8). To aid visualization of the twin boundary shown in Fig. 8, a black line, which also defines the {101} surface, is drawn perpendicular to the [101] direction and represents the core of the grain boundary. This image supports the experimental finding (HAADF images) showing no displacements of A-site and B-site columns. The orthorhombic unit cell arises from rotation of the Mn octahedra, which causes them to rotate down or up. This can be easily viewed in terms of shape of the window of the A cation site in the [101] direction, which is diamond-shaped, arranged vertically (1) or horizontally (2) (Fig. 8(a)). Fig. 8(a) shows the domain boundary along the [101] direction and indicates that when the two surfaces ({101} and {–101}) come into contact, the alternating pattern (1)–(2) cannot be imposed at the boundary region indicated by the double black arrow, and the Mn octahedra will have to rotate to accommodate the distortion, resulting in distorted windows (1)–(2) near the core of the grain boundary, where the windows are almost squared due to the Mn octahedra almost aligned (distorted (3)). Again to aid visualization, these diamond shaped windows are drawn in the figure; there are 3 distorted windows at the grain boundary that cannot be matched with the bulk like windows in black (1) and red (2). Fig. 8(b) represents the domain boundary along the [010] direction and reiterates the experimental findings indicating that the different BF contrast is related only to the change in the oxygen positions for the two [101]_ortho_ and [–101]_ortho_ planes. As shown in Fig. 8(c), which represents the surface {101} along the [010] direction, the Mn octahedra form a repeating pattern up–down (UP–DN) whether or not they rotate in opposite directions. However, looking at the domain boundary orientated in the same [010] direction (Fig. 8(b)), this pattern is lost. The relaxation of the Mn octahedra, due to the two joint surfaces, imposes a distortion for approximately 1 nm either side of the boundary core. This forces six Mn octahedra to retain the same orientation, 6 UP or 6 DN as shown in Fig. 8(b).

**Fig. 9:**
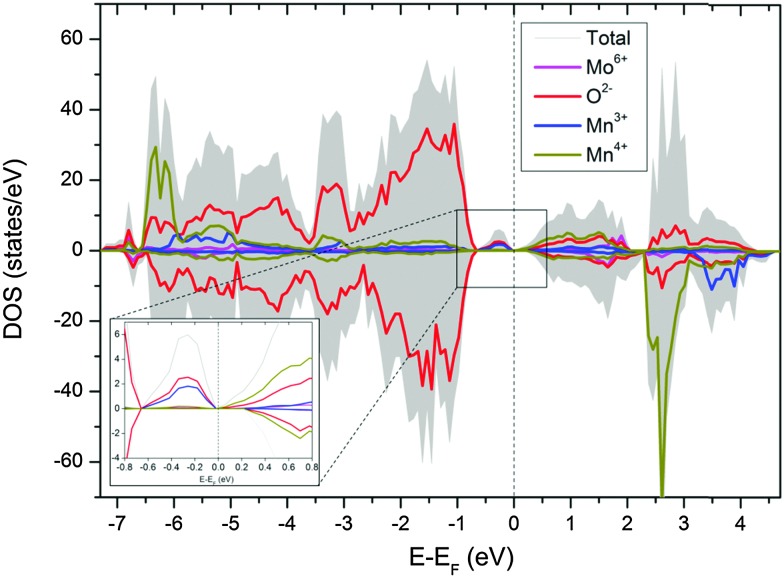
Partial density of state of CaMn_1–3*x*_^4+^Mn_2*x*_^3+^Mo_*x*_^6+^O_3_. The inset shows the states in the vicinity of the Fermi level.

Substitution of Mo in the B-site will promote a mixed valance state for Mn according to CaMn_1–3*x*_^4+^Mn_2*x*_^3+^Mo_*x*_^6+^O_3_. Therefore, substitution of *x* mol% of Mo to the B-site will generate 2*x* mol% of Mn^3+^, which is 8 mol% Mn^3+^ for compositions with 4 mol% substitution of Mo (*i.e.* composition CMM, C9S1MM4, C7S3MM4 and C4S6MM4). DFT calculations demonstrate the effect of Mo substitution by showing that the Fermi level is dominated by Mn^3+^ and O^2–^ states (Fig. 9), which appear only upon Mo substitution on Mn sites. The original composition (Ca_0.6875_Sr_0.3125_Mn_0.9375_Mo_0.0625_O_3_) can therefore be rewritten as Ca_0.6875_Sr_0.3125_Mn_0.8125_^4+^Mn_0.125_^3+^Mo_0.0625_^6+^O_3_. Hence, the effect of Mo substitution is only to increase the concentration of electronic carriers, which in turn affects the thermoelectric properties of the material.

**Fig. 10:**
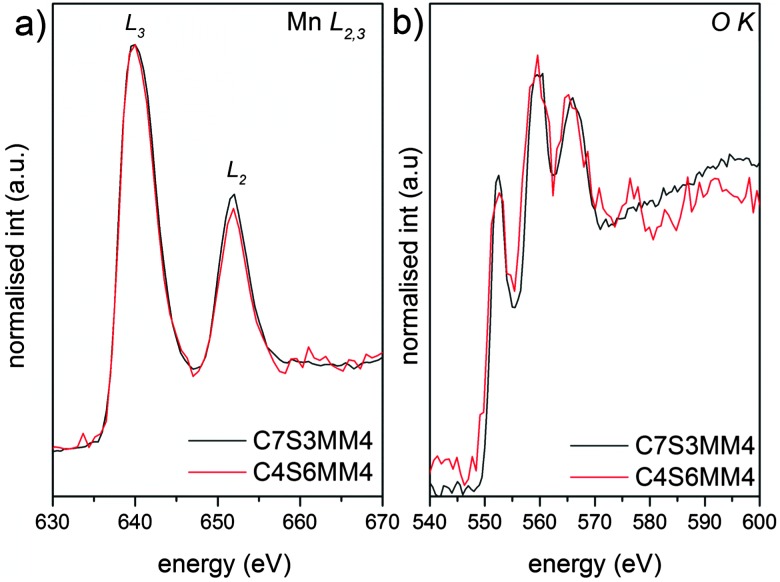
Background subtracted (a) Mn L_2,3_ and (b) O K EELS spectra of C7S3MM4 (black) and C4S6MM4 (red) compositions.

The EELS L edges of transition metals such as manganese result from the excitation of 2p electrons into empty bound states or the continuum. Thus, these edges show two characteristic white lines, originating from transitions from the spin orbit split 2p_3/2_ and 2p_1/2_ levels to the available states in the 3d band. It has been shown that the intensity ratio of the L_2_ and L_3_ white lines is a characteristic of the oxidation state of manganese ions.^53^ Fig. 10 shows the Mn L_2,3_ and O K edges for ceramic C7S3MM4. Using the same type of curve-fitting analysis as used by Varela *et al.* ^54^ to obtain the valence state of Mn in Ca_1–*x*_La_*x*_MnO_3_, the white-line intensity for C7S3MM4 and C6S4MM4 was found to be 2.1 ± 0.1 and 2.0 ± 0.1, respectively. This suggests the Mn valence state to be close to 4+ for both samples.^53^ Considering the 10% uncertainty in the data from this technique it is difficult to accurately define the Mn^3+^ content of the sample. However, electrical conductivity data which will be discussed later suggests the formation of Mn^3+^ due to Mo substitution in the B-site. Bocher *et al.* ^16^ in an attempt to determine the Mn valence state in Nb doped CaMnO_3_ reported a small shift in the binding energy of the Mn^4+^ oxidation state in Nb substituted CaMnO_3_ and suggested the formation of Mn^3+^. However, the reported values of the shift are too small, and the shift could equally well be due to a change in Mn^4+^ binding energy (resulting from a change of composition) as well as a change in the valence state of Mn. Unfortunately, neither EELS nor XPS provide unambiguous data for these materials.

### Thermoelectric properties

**Fig. 11:**
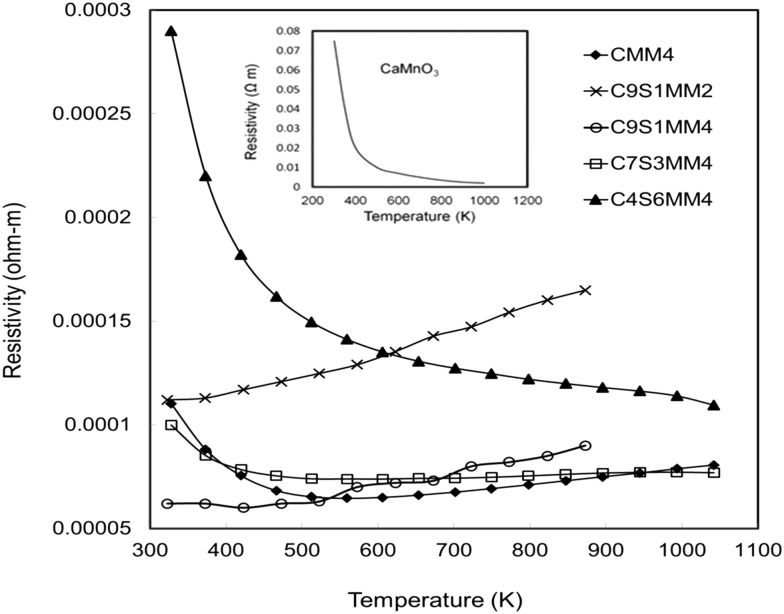
Temperature dependence of the electrical resistivity of Sr–Mo substituted CaMnO_3_. Data for undoped CaMnO_3_ is shown in the inset (adopted from Xu *et al.* ^15^).

The resistivity data for the full set of experimental samples are presented in Fig. 11. Data for undoped CaMnO_3_, adopted from the work of Xu *et al.* ^15^ is shown in the inset of the figure. The resistivity values for the Sr–Mo doped samples are in the range from 3.8 × 10^–4^ Ωm to 5 × 10^–5^ Ωm in the temperature window of measurement (300 K to 1000 K). The resistivities for compositions C9S1MM4, C7S3MM4 are amongst the lowest values reported for B-site doped CaMnO_3_.^15,16^ The substitution of 4 mol% Mo for Mn (composition CMM4) reduces the resistivity by a factor of 3–4. This is due to the creation of additional charge carriers due to the presence of Mn^3+^ in the Mn^4+^ matrix.^13^ The CMM4 sample shows a metallic behaviour above 400 K with values of approximately 1 × 10^–4^ Ω m in the temperature range 400–1000 K. The resistivity values and behaviour of compositions C9S1MM4 and C3S7MM4 are similar to that of CMM4. The composition with the highest Sr content, C4S6MM4 showed the highest resistivity at lower temperatures with semi-conducting behaviour. This increase in the resistivity for C4S6MM4 is due to the disordered distribution of Sr and Ca in the A-sites (as shown in the electron microscopy section) reducing charge mobility. Nevertheless, the low resistivity of C9S1MM4 and C7S3MM4 was aided by high density and Mo content. It was not possible to prepare high density, crack-free samples of CMM4.

**Fig. 12:**
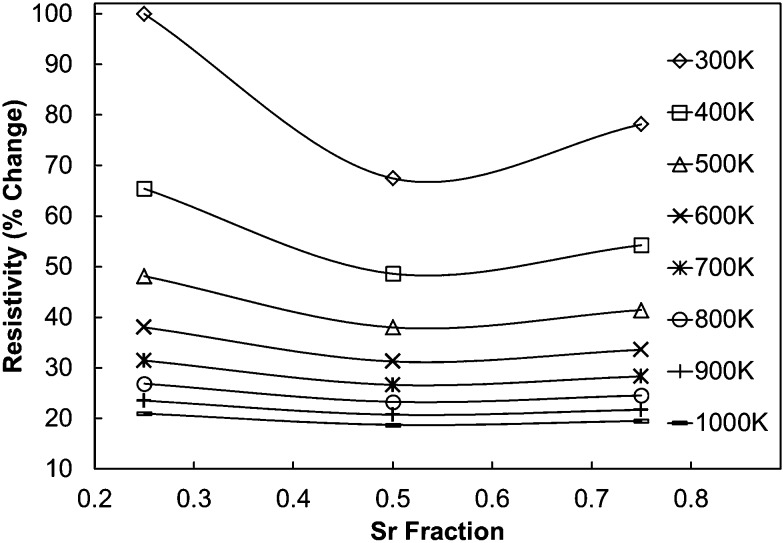
Calculated percentage change in resistivity of (Ca,Sr)MnO_3_ samples with respect to C75S25MO, which shows the highest resistivity at room temperature according to DFT calculations.

As the experimentally determined resistivity data suggested a broadly intrinsic behaviour, with samples exhibiting either semiconducting or metallic characteristics, we have performed DFT calculations on simple systems to decouple the effect of co-doping (with Mo and Sr) on the resistivity. As the calculated resistivity values are obtained with respect to the relaxation time, which is an unknown parameter, we present the percentage change in resistivity with respect to C7.5S2.5MO (Fig. 12), which showed the highest resistivity at room temperature. All the data for computational samples show a semiconducting behaviour with the resistivity decreasing with increasing temperature. Indeed, the resistivity at high temperature is similar for all compositions, while intermediate Sr doping gives the lowest resistivity at low temperature. It is more likely, however, that the resistivity will not be strongly affected by aliovalent Sr substitution. Instead, we infer that the microstructure and reduction of Mn ions will be the more likely cause of this complicated behaviour. In our previous work,^23^ we have indeed shown that higher concentrations of Mn^3+^ decrease the resistivity, but this interpretation is not straightforward as the behaviour is strongly influenced by the arrangement of oxygen vacancies (*V*_o_) (in line with the discussion in Loshkareva *et al.* ^55^), which are unavoidable intrinsic defects in perovskite materials.^20^ As noted previously, Mo doping will have a similar effect as *V*_o_ doping, as both defects have been shown to have the effect of increasing the concentration of Mn^3+^. Consequently, an increase of Mo in the experimental samples leads to a reduction in resistivity, with resistivity of C9S1MM2 greater than that of C9S1MM4.

**Fig. 13:**
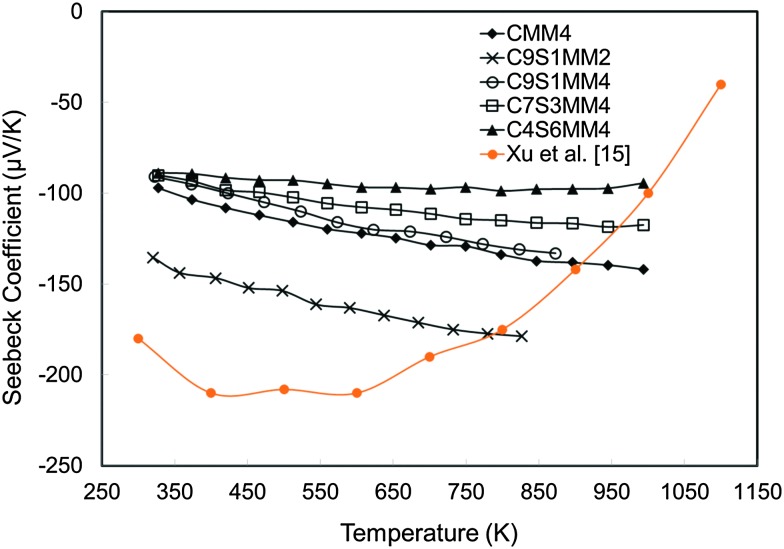
Experimentally determined Seebeck coefficients of Sr–Mo substituted CaMnO_3_ as a function of temperature.

**Fig. 14:**
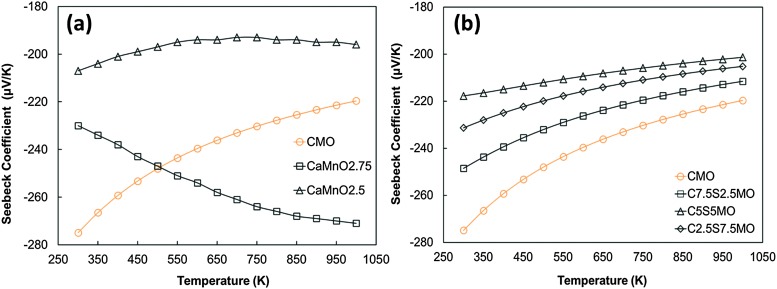
Calculated temperature dependence of Seebeck coefficient of (a) defective CaMnO_3–*δ*_ (redrawn from Molinari *et al.* ^23^) and (b) Sr substituted CaMnO_3_.

The experimental Seebeck coefficient data are presented in Fig. 13. All Seebeck coefficients are negative, indicating n-type conduction, and increase uniformly with increasing temperature. At low temperatures, the values are smaller than those reported by Xu *et al.* ^15^ for undoped CaMnO_3_ (Fig. 13). Increasing Sr substitution for Ca reduced the Seebeck coefficients, but the materials still exhibited moderate values of –90 to –190 μV K^–1^ at 1000 K. The highest Seebeck coefficient was obtained for C9S1MM2 which contained 2 mol% Mo; further increase in the Mo content led to a reduction in the Seebeck coefficient as a result of changes in carrier concentration. These trends are in agreement with our DFT calculations (Fig. 14). In particular, the calculations showed a reduction of the Seebeck coefficient (i) with the increasing concentration of Mn^3+^ ^23^ (Fig. 14a), and (ii) with the increasing Sr content – the highest values are found for samples with the lowest Sr content (Fig. 14b). Our data also show that intermediate doping levels do not improve the Seebeck coefficients, while high (75%) and low (25%) Sr substitutions do. The calculated temperature dependence for the Seebeck coefficient of Sr substituted samples, as well as for stoichiometric CaMnO_3_, show a decrease in the value with increasing temperature, while the oxygen sub-stoichiometric samples show different trends (Fig. 14a and b) more in line with experimental findings. It is clear that changes in the measured Seebeck coefficients arise from the complex experimental structure and thus our DFT data can be used as a guideline to disentangle the effects of Sr and Mn^3+^ doping.

It has been shown that in complex mixed Mn^3+^/Mn^4+^ manganates, such as these, Jahn–Teller distortions (JT) can have an impact on the Seebeck coefficient^56–58^ and should therefore be considered. The Seebeck coefficient (*S*) may be calculated using the Heikes formula,^56^ involving only the electronic degeneracy of Mn^3+^ and Mn^4+^ species and the molar fraction:
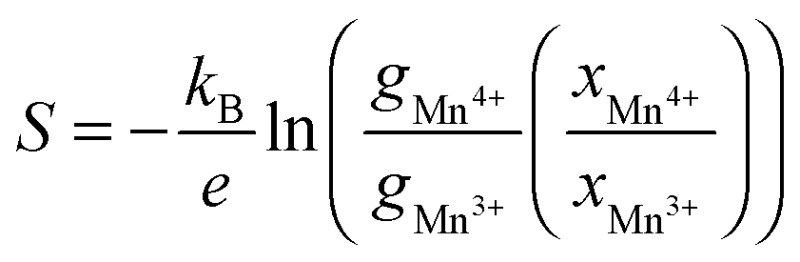
where *k*_B_ is the Boltzmann constant, *e* is the electronic charge, *x* is the molar fraction of the species, and *g*_Mn^3+^_ and *g*_Mn^4+^_ are the electronic degeneracy of the electron donor Mn^3+^ and the electron acceptor Mn^4+^ (their values will depend on the spin and JT distortion). The ratio between the molar fraction of Mn^4+^ (number of available sites for hopping charges) and Mn^3+^ does not account for the molar faction of octahedral environments occupied by Mo^6+^ as these sites are not available for electron hopping. We have assumed that *x*_Mn^3+^_ is double *x*_Mo^6+^_ as each Mo^6+^ injects two electrons in the system. By considering electronic degeneracy (product of spin and orbital degeneracy) for Mn^4+^, Mn^3+^, and Mn^3+^ with Jahn–Teller (JT) distortion (degenerate t_2g_ orbitals and split e_g_ orbitals), for appropriate high spin (HS) and low spin (LS) configurations, we have calculated the Seebeck coefficients for experimental formulations using the Heikes formula. The results are presented in Table 7.

**Table 7:** Seebeck coefficient calculated using the Heikes (*S*_H_) formula considering Mn^4+^ always in a HS state and Mn^3+^ in LS, HS and JT. The *S*_Expt_: *T* → ∞ (low *T*) was calculated from the experimentally measured values of the Seebeck coefficient, extrapolated to infinite *T* including only values below 500 K as in this region the electrical conductivity is relatively low. The *S*_Expt_: *T* → ∞ (high *T*) was calculated from the experimentally measured values of the Seebeck coefficient, extrapolated to infinite temperature including only values above 700 K (Extrapolated values taken from *S* values in Fig. 13.)

Formulation	*S*_H_ (μV K^–1^) (Mn^4+^, HS/Mn^3+^, LS)	*S*_H_ (μV K^–1^) (Mn^4+^, HS/Mn^3+^, HS)	*S*_H_ (μV K^–1^) (Mn^4+^, HS/Mn^3+^, JT)	*S*_Expt_ (μV K^–1^) *T* → ∞ high *T*	*S*_Expt_ (μV K^–1^) *T* → ∞ low *T*
CaMn_0.96_Mo_0.04_O_3_	–137	–128	–187	–175	–147
Ca_0.9_Sr_0.1_Mn_0.98_Mo_0.02_O_3_	–202	–193	–253	–210	–188
Ca_0.9_Sr_0.1_Mn_0.96_Mo_0.04_O_3_	–137	–128	–187	–177	–134
Ca_0.7_Sr_0.3_Mn_0.96_Mo_0.04_O_3_	–137	–128	–187	–134	–124
Ca_0.4_Sr_0.6_Mn_0.96_Mo_0.04_O_3_	–137	–128	–187	–92	–102

Comparing the extrapolated experimental values from the region of low *T* with the expected high temperature limit of the Seebeck coefficients according to the Heikes formula, we would expect that the Mn^3+^ ions should be in the low or high spin states and there is no evidence for Jahn–Teller distortion. Similarly, comparing the extrapolated experimental values from the region of high *T* with the expected high temperature limit of the Seebeck coefficients according to the Heikes formula, we would expect that only samples that do not contain Sr substitution on the A site will contain Mn^3+^ ions with Jahn–Teller distortion, while samples containing Sr substitution do not (with the exception of C9S1MM4). An effect on the Seebeck coefficient due to Sr substitution was indeed determined by using DFT calculations, where we found a reduction of the Seebeck coefficient with increasing Sr content, also in agreement with the values of *S*_Expt_. in Table 7.

Structural data can also be used to detect the presence of Jahn–Teller distortion. Calculated Mn–O distances (from the experimental data, Table 5) differ by less than 0.007 Å between Mn–O_eq_ and Mn–O_ap_ for all samples with the exception of C9S1MM4 and CMM4 where the differences are 0.02 and 0.01 Å, respectively. Whether these differences are meaningful is debatable as the experimental samples are polycrystalline. However, DFT calculations do support the proposal of Jahn–Teller distortion in octahedral sites containing Mn^3+^, but not in octahedral sites containing Mn^4+^. Thus, it is not possible to rule out the presence of Jahn–Teller distortion from the experimental data but we support its presence from the computational data.

**Fig. 15:**
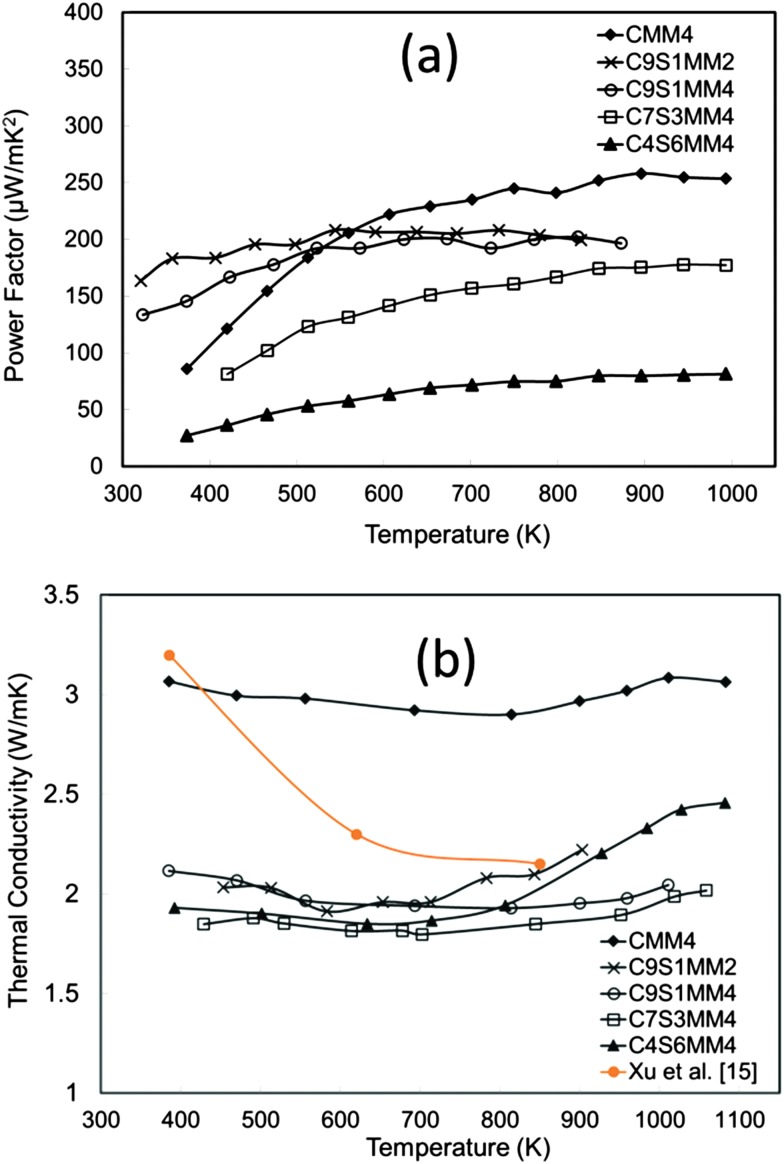
(a) Temperature dependence of the experimental power factor of Sr–Mo substituted CaMnO_3_, (b) temperature dependence of thermal conductivity of Sr–Mo substituted CaMnO_3_.

The power factor data are presented in Fig. 15(a). The power factor values are in the range reported for both A-site and B-site substituted CaMnO_3_.^11,12,14,18^ It can be seen that substitution of Sr reduces the power factor. This is mainly due to reduction of the Seebeck coefficient as discussed in the above section.

The thermal conductivity data are presented in Fig. 15(b). Interestingly, thermal conductivity is much reduced by Sr doping, and the values are lower than for undoped CaMnO_3_ (data from Xu *et al.* ^15^). The introduction of Sr with a larger ionic radius than Ca induces structural modification, reducing phonon propagation. Similar effects for A-site doped CaMnO_3_ have been reported by Ohtaki *et al.* ^14^ and Dabrowski.^59^

The electronic thermal conductivity, *k*_el_, was calculated using the formula:*k*_el_ = *L*_0_ *Tδ*where *L*_0_ is the Lorenz factor (2.44 × 10^–8^ W Ω K^–2^) and *δ* is the conductivity of the sample at temperature *T*. The calculated *k*_el_ values were significantly higher for doped samples compared to CaMnO_3_. This can be attributed to improved conductivity of the samples. However, even for doped samples the contributions of *k*_el_ were less than 10% of overall thermal conductivity. Hence, suppression of lattice thermal conductivity, *k*_lattice_ can be achieved by introducing A/B site dopants of different ionic sizes, and the presence of sub-grains/domains, and twins all lead to reduction of the total thermal conductivity (*k*_total_) in CaMnO_3_ based materials. The sample C7S3MM4 exhibits the lowest *K*_total_ of 1.6 W m^–1^ K^–1^ at 1000 K.

**Fig. 16:**
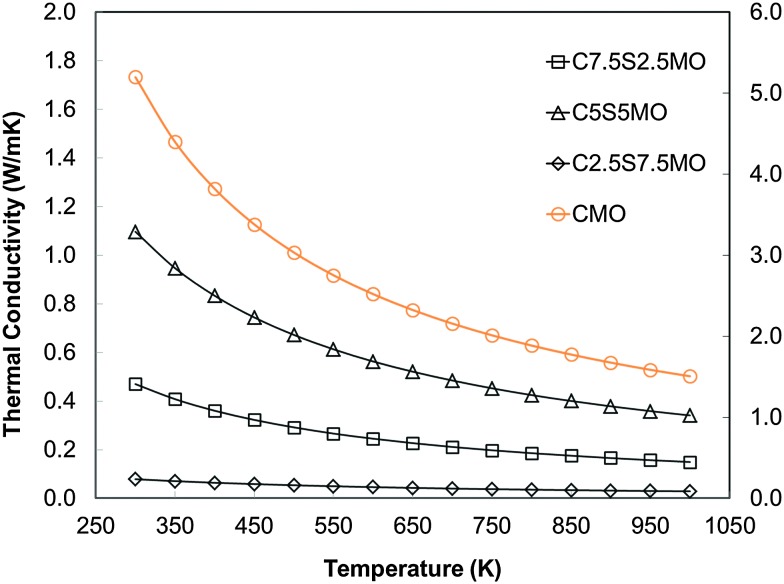
Calculated temperature dependence of lattice thermal conductivity (*k*_lattice_) of Sr substituted CaMnO_3_.

Whether or not Sr substitution reduces *k*_lattice_, is hard to evaluate from experimental data alone, where the sample microstructure and complex defects play important roles. Thus, our DFT calculations on single bulk crystals are crucial to determine the effect of Sr substitution on thermal transport in the perovskite structure. Our results show clearly that Sr substitution reduces *k*_lattice_ compared to that in pure CaMnO_3_ (Fig. 16). However the trend and effect is not directly proportional to the Sr content; the lowest concentration of the dopant is more beneficial than the intermediate concentration. The calculations demonstrate that introducing 25% of a different cation on the perovskite A site, decreases *k*_lattice_ by increasing phonon–phonon scattering.

In order to assess whether the presence of domain boundaries directly affects *k*_lattice_ we used molecular dynamics, which enabled us to use large simulation cells. The calculated thermal conductivity of CaMnO_3_ containing two domain boundaries spaced 7.5 nm apart was found to be reduced by approximately 7% compared to the stoichiometric single crystal. This reduction is clearly dependent on the concentration of these domain boundaries and their distance apart. A detailed computational quantitative evaluation of the impact of domain boundaries and related structural features on *k*_lattice_ would therefore be very valuable but it is beyond the scope of this paper.

**Fig. 17:**
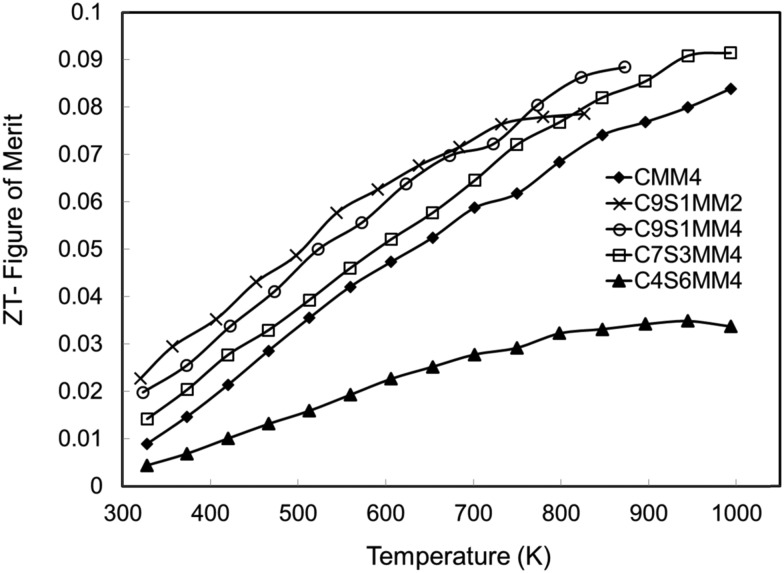
*ZT* values for Sr–Mo substituted CaMnO_3_.

The *ZT* data are presented in Fig. 17. Consistent with other investigations of CaMnO_3_ based materials,^15,16,18^ the *ZT* values increase almost linearly with temperature. Whilst the samples rich in Sr and Mo (C4S6MM4) exhibit the lowest *ZT* values, the data for the other samples are clustered together (Fig. 17), with C9S1MM2 having significantly higher values up to 800 K. The superior properties of C9S1MM2 arise from the combination of lower resistivity (Fig. 11) and higher Seebeck coefficients (Fig. 13). The addition of Mo to the perovskite is beneficial to the thermoelectric performance because of the generation of Mn^3+^ and additional carriers. Whilst the *ZT* values for Mo-added CaMnO_3_ (CMM4) are much superior to those of undoped CaMnO_3_, the best properties were obtained for materials containing modest amounts of both Sr (on the A-site), and an optimum addition of Mo (2 mol%). Higher levels of Mo led to a reduction in the Seebeck coefficient and thereby the power factor and *ZT*.

The thermoelectric properties of CaMnO_3_-based ceramics depend critically on the changes in the composition, structure and transport behaviour as demonstrated by high resolution electron microscopy and modelling. Substitution of Sr stabilises the tetragonal form of CaMnO_3_; atomically resolved imaging and analysis showed a random distribution of Sr in the A-site of the perovskite structure and revealed a boundary structure of 90° rotational twin boundaries across {101}_orthorhombic_; the latter are predominant phonon scattering sources to lower the thermal conductivity as suggested by potential based calculations. Simultaneous HAADF and BF images provided experimental evidence for changes in the oxygen positions for the two [101]_ortho_ and [–101]_ortho_ planes. Substitution of Mo on the B site leads to the generation of additional carriers due to the presence of Mn^3+^ in the Mn^4+^ matrix. This reduces electrical resistivity, but excess amounts of Mo also reduced the Seebeck coefficients. Introducing A/B site dopants of different ionic sizes increased phonon scattering, thereby reducing thermal conductivity. The different types of sub-grain structure (wavy and straight sub-grain boundaries) domains boundaries and twins (revealed by electron microscopy) also help to reduce thermal conductivity. The importance of such features was highlighted by potential based molecular dynamics that showed the presence of two domain boundaries spaced 7.5 nm apart in CaMnO_3_ could reduce the thermal conductivity by approximately 7% compared to the stoichiometric single crystal. Ceramics of Ca_0.7_Sr_0.3_Mn_0.96_Mo_0.04_O_3_ exhibit enhanced properties with *S*_1000K_ = –180 μV K^–1^, *ρ*_1000K_ = 5 × 10^–5^ Ωm, *k*_1000K_ = 1.8 W m^–1^ K^–1^ and *ZT* ≈ 0.11 at 1000 K.

## Conclusions

High quality, crack-free CaMnO_3_-based ceramics, with densities greater than 90% theoretical, were produced by addition of Sr, and slow cooling rates after sintering. Substitution of Ca by Sr in CaMnO_3_ changes the symmetry from orthorhombic (*Pnma*) to tetragonal (*I*4/*mcm*); the larger Sr ions stabilise the tetragonal structure by broadening the structural transition window, thereby improving material stability.

Cooling from the high temperature cubic form during sintering gives rise to sub-grain boundaries within the grains which are predominantly wavy in Sr-poor (orthorhombic symmetry) materials, and straight in the Sr-rich (tetragonal symmetry) ceramics.

There was no evidence of Sr and Ca ordering in the A-site of the perovskite structure, but atomic resolution microscopy suggested that differences in bright field images are related to changes in the oxygen positions for the two [101]_ortho_ and [–101]_ortho_ planes. Modelling supported the experimental findings that there was no lateral displacement of A-site and B-site columns.

The presence of Mo in the initial powder formulation is beneficial for processing, but its incorporation in the final ceramic increases the concentration of electronic carriers, improving thermoelectric properties. DFT calculations demonstrated that the Fermi level was dominated by Mn^3+^ and O^2–^ states upon Mo substitution. Furthermore, DFT calculations supported the presence of Jahn–Teller distortion of octahedral sites containing Mn^3+^.

Electrical resistivity values for the Mo doped samples are amongst the lowest reported for B-site doped CaMnO_3_. This arises from the creation of additional charge carriers due to the presence of Mn^3+^ in the Mn^4+^ matrix. The higher resistivity in the Sr–Mo doped samples appears to be related to the disordered distribution of Ca and Sr on A sites, and possibly the arrangement of oxygen vacancies.

High levels of Sr and Mo were detrimental to the Seebeck coefficient, as a result of changes in carrier concentrations, as confirmed by DFT calculations. However, substituting Sr for Ca in CaMnO_3_ causes structural modifications, and the presence of A/B site dopants of different ionic sizes, and the existence of sub-grains/domains, and twins leads to a reduction in the total thermal conductivity. DFT calculations show that Sr substitution reduces *k*_lattice_ compared to that in pure CaMnO_3_, while potential based molecular dynamics calculations showed that the presence of two close domain boundaries will have a significant impact on phonon transport, reducing thermal conductivity.
