# Supplementary material for: Crystal structure and thermoelectric properties of Sr–Mo substituted CaMnO3: a combined experimental and computational study
Source: J Mater Chem C Mater. 2015 Nov 13;3(47):12245–59. doi: 10.1039/c5tc02318a (PMC5361175; doi:10.1039/c5tc02318a)
Supplement: Supplementary file 1 [file TC-003-C5TC02318A-s001.pdf]

Electronic Supplementary Material (ESI) for Journal of Materials Chemistry C.
This journal is © The Royal Society of Chemistry 2015

Crystal Structure and Thermoelectric Properties of Sr - Mo Substituted $CaMnO_3$: A Combined Experimental and Computational Study

D. Srivastava [1], F. Azough [1], R. Freer [1]

E. Combe[2], R. Funahashi[2]

D.M. Kepaptsoglou [3], Q.M. Ramasse[3]

M. Molinari[4], S. R. Yeandel[4], J. D. Baran[4], S. C. Parker[4]

[1] School of Materials, University of Manchester, Manchester, M13 9PL, U.K.

[2]National Institute of Advanced Industrial Science and Technology, Midorigaoka, Ikeda, Osaka 563-8577, Japan

[3] SuperSTEM Laboratory, STFC Daresbury Campus, Daresbury WA4 4AD, U.K.

[4]Department of Chemistry, University of Bath, Claverton Down, Bath BA2 7AY, U.K.

Supplementary Data

S1. Refinement of X-Ray Diffraction data

The X-ray diffraction spectra were refined using TOPAS software. The instrument parameters were corrected for the diffractometer used; Cif files were downloaded from ICSD database for CaMnO3 compositions with different spacegroups: Pnma and I4/mcm as initial structures. The atomic substitutions were taken into account and atomic positions were refined while occupancy was fixed according to the stoichiometry of the compositions prepared. The B_{eq} parameters were minimized during refinement. The measured, calculated pattern and residual are presented for each composition in Figure S1. The peaks are indexed in the main paper.

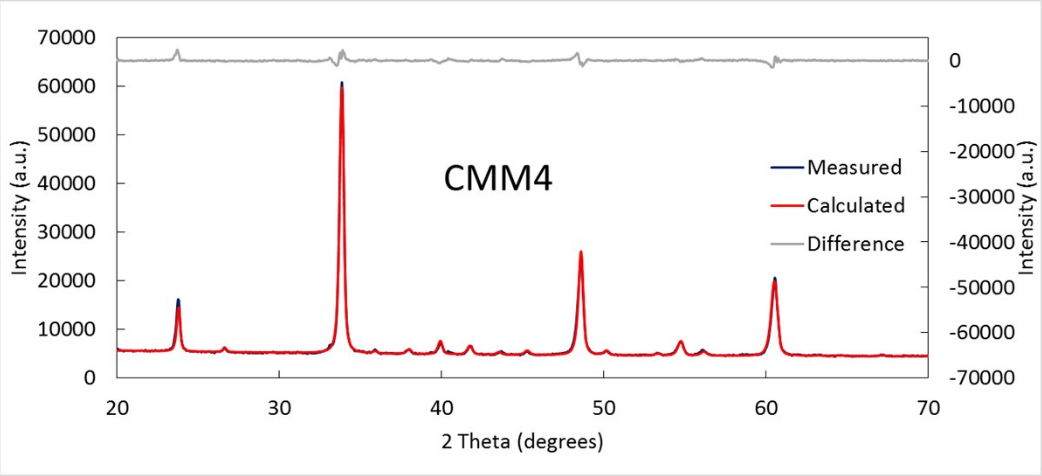
CMM4
Measured
Calculated
Difference
Intensity (a.u.)
Intensity (a.u.)
70000
60000
50000
40000
30000
20000
10000
0
0
-10000
-20000
-30000
-40000
-50000
-60000
-70000
20
30
40
50
60
70
2 Theta (degrees)

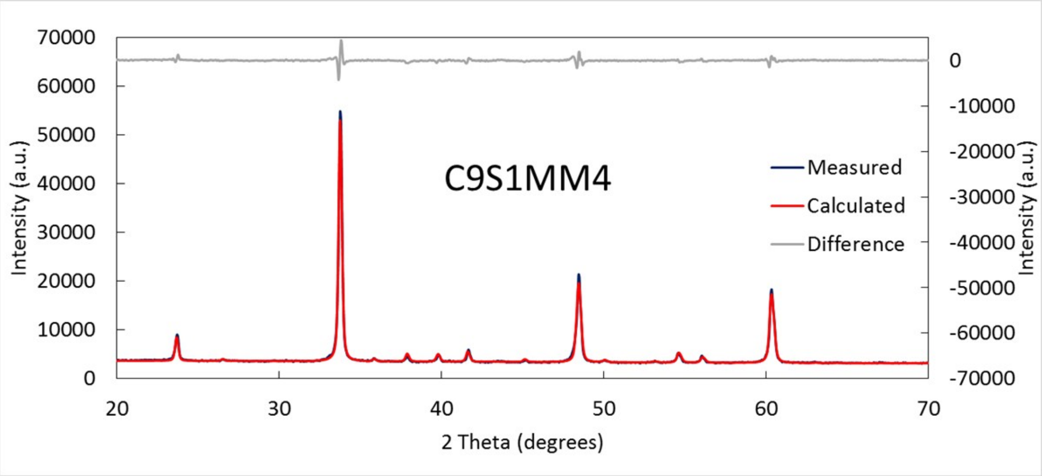
C9S1MM4
Measured
Calculated
Difference
Intensity (a.u.)
Intensity (a.u.)
70000
60000
50000
40000
30000
20000
10000
0
0
-10000
-20000
-30000
-40000
-50000
-60000
-70000
20
30
40
50
60
70
2 Theta (degrees)

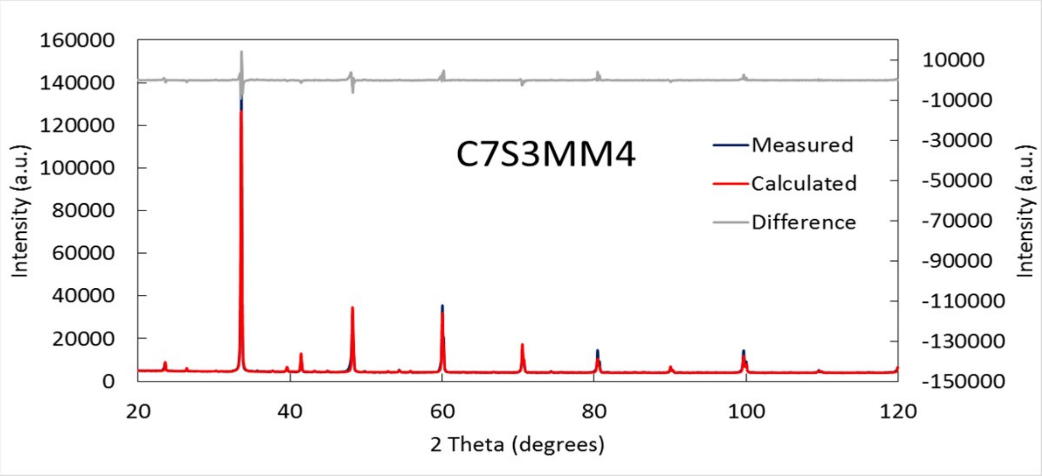
C7S3MM4
Measured
Calculated
Difference
Intensity (a.u.)
Intensity (a.u.)
160000
140000
120000
100000
80000
60000
40000
20000
0
10000
-10000
-30000
-50000
-70000
-90000
-110000
-130000
-150000
20
40
60
80
100
120
2 Theta (degrees)

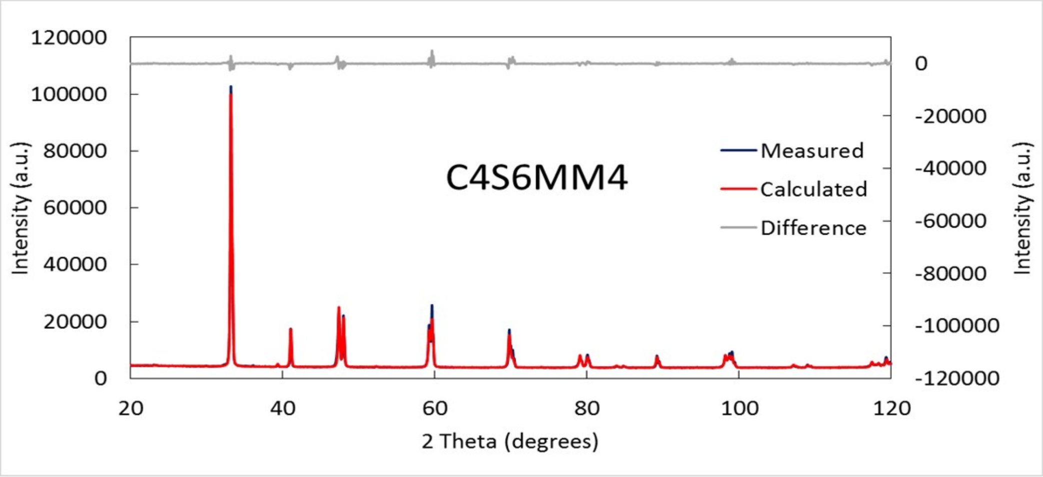

Figure S1 Refined XRD spectra obtained using TOPAS

S2. Heat Capacity Measurements

The measurement were carried out using a Netzsch STA 449c, with a heating rate of 20 K/min and Sapphire as standard sample. The uncertainty in reported values is ± 10%.

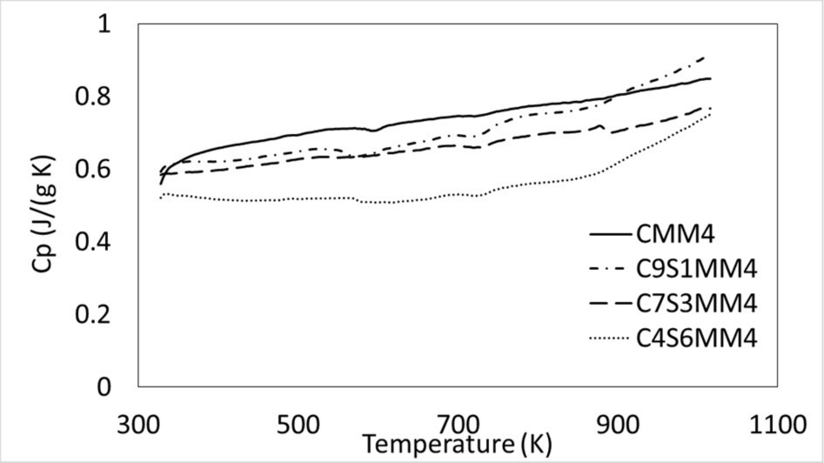

Figure S2. Temperature dependence of heat capacity for Calcium Manganate based samples indicating the effect of Sr substitution.
